# Retracted: Correlation between High Myopia Susceptibility and Polymorphisms of RASGRF1 Gene among College Students in Zhejiang

**DOI:** 10.1155/2023/9814180

**Published:** 2023-12-13

**Authors:** Journal of Environmental and Public Health

This article has been retracted by Hindawi, as publisher, following an investigation undertaken by the publisher [1]. This investigation has uncovered evidence of systematic manipulation of the publication and peer-review process. We cannot, therefore, vouch for the reliability or integrity of this article.

Please note that this notice is intended solely to alert readers that the peer-review process of this article has been compromised.

Wiley and Hindawi regret that the usual quality checks did not identify these issues before publication and have since put additional measures in place to safeguard research integrity.

We wish to credit our Research Integrity and Research Publishing teams and anonymous and named external researchers and research integrity experts for contributing to this investigation.

The corresponding author, as the representative of all authors, has been given the opportunity to register their agreement or disagreement to this retraction. We have kept a record of any response received.

## References

[1] Yi J., Dai Y., Ma S., Zheng Y., Liang Y., Huang X. (2023). Correlation between High Myopia Susceptibility and Polymorphisms of RASGRF1 Gene among College Students in Zhejiang. *Journal of Environmental and Public Health*.

